# The Potential of Stilbene Compounds to Inhibit M^pro^ Protease as a Natural Treatment Strategy for Coronavirus Disease-2019

**DOI:** 10.3390/cimb45010002

**Published:** 2022-12-20

**Authors:** Ayesha Naseem, Fatima Rasool, Abrar Ahmed, Wayne G. Carter

**Affiliations:** 1 Faculty of Pharmacy, Punjab University College of Pharmacy, University of the Punjab, Lahore 54590, Pakistan; 2 School of Medicine, University of Nottingham, Royal Derby Hospital Centre, Derby DE22 3DT, UK

**Keywords:** astringin, COVID-19 disease, isorhapontin, isorhapontigenin, piceatannol, pinosylvin, pinosylvin monomethyl ether, SARS-CoV-2 replication inhibitor, stilbene, resveratrol

## Abstract

COVID-19 disease has had a global impact on human health with increased levels of morbidity and mortality. There is an unmet need to design and produce effective antivirals to treat COVID-19. This study aimed to explore the potential ability of natural stilbenes to inhibit the M^pro^ protease, an acute respiratory syndrome coronavirus-2 (SARS-CoV-2) enzyme involved in viral replication. The binding affinities of stilbene compounds against M^pro^ were scrutinized using molecular docking, prime molecular mechanics-generalized Born surface area (MM-GBSA) energy calculations, and molecular dynamic simulations. Seven stilbene molecules were docked with M^pro^ and compared with GC376 and N3, antivirals with demonstrated efficacy against M^pro^. Ligand binding efficiencies and polar and non-polar interactions between stilbene compounds and M^pro^ were analyzed. The binding affinities of astringin, isorhapontin, and piceatannol were −9.319, −8.166, and −6.291 kcal/mol, respectively, and higher than either GC376 or N3 at −6.976 and −6.345 kcal/mol, respectively. Prime MM-GBSA revealed that these stilbene compounds exhibited useful ligand efficacy and binding affinity to M^pro^. Molecular dynamic simulation studies of astringin, isorhapontin, and piceatannol showed their stability at 300 K throughout the simulation time. Collectively, these results suggest that stilbenes such as astringin, isorhapontin, and piceatannol could provide useful natural inhibitors of M^pro^ and thereby act as novel treatments to limit SARS-CoV-2 replication.

## 1. Introduction

The Coronavirus-2019 (COVID-19) disease is a global pandemic that has impacted human health with increased levels of morbidity and mortality. The COVID-19 disease outbreak originated in Wuhan, the capital city of the Hubei province of China, in 2019, with initially high mortality of approximately 3% of infected individuals, a relatively high mortality that likely reflected limitations of healthcare provision at the epicenter of the outbreak [1]. Subsequently, the World Health Organization (WHO) declared that the COVID-19 disease, caused by the severe acute respiratory syndrome coronavirus-2 (SARS-CoV-2) virus, was a global pandemic. SARS-CoV-2, a single positive-stranded RNA virus, causes respiratory disease and is transmitted between humans primarily by direct modes via inhalation of viral (aerial) droplets passed from talking, coughing, or sneezing [2,3]. The COVID-19 disease is highly contagious and generates symptoms ranging from asymptomatic to mild to moderate and severe respiratory distress, pneumonia, and multiple organ failure [2,3]. Contracting COVID-19 disease can also have a serious psychological impact on patients with an increased likelihood of suffering from depression, stress, anxiety, and/or post-traumatic stress disorder (PTSD) [4,5,6].

The development of vaccines and their implementation has proved effective at reducing mortality, but as of May 2022, almost one billion people in lower-income countries remain unvaccinated [3,7,8]. Therapeutic drugs have also been developed and previously marketed drugs repurposed as COVID-19 treatments, including antivirals, antimalarials, and broad-spectrum antibiotics, as well as immunotherapy approaches adopted, including anti-inflammatory drugs and targeted monoclonal antibodies [3,8,9].

The SARS-CoV-2 genome has 11 open reading frames (ORFs), the first of which (ORF 1a/b) is translated into two polyproteins: pp1a and pp1ab, and both are processed by the main protease M^pro^ and papain-like protease (PL^pro^), to generate 16 non-structural proteins (nsps), some of which contribute to viral replication [10]. Therefore, blocking the activity of proteins involved in the generation of the nsps (M^pro^ and PL^pro^) will likely hinder viral replication efficiency. Hence, M^pro^ has been investigated as a drug target for repurposed and rationally and artificially intelligently designed inhibitors, including GC376 and N3 [11,12,13,14,15,16,17,18].

Primary or secondary metabolites of medicinal plants can also act as potential lead compounds against SARS-CoV-2 target proteins, including M^pro^ [19,20,21]. Natural stilbenes are phytochemicals characterized by the presence of a 1,2-diphenylethylene nucleus and can be monomeric or oligomeric compounds [22]. A wide variety of plant species produce stilbene compounds as secondary metabolites [22,23], and they are also present in fruits, including grapes, as well as within tree bark [22,23,24]. Stilbenes have diverse and beneficial biological activities in addition to their inherent ability to act as antioxidants to combat cellular redox stress [25,26]. In silico data has suggested that a dimeric stilbene skeleton could provide a useful structural basis for the development of drugs to counter COVID-19 disease [27].

Hence, this manuscript aimed to consider the potential use of natural stilbene inhibitors to halt SARS-CoV-2 viral replication via binding to M^pro^. Seven stilbene molecules were docked with M^pro^, and their binding affinities were compared with two reference ligands, GC376 and N3. Prime molecular mechanics-generalized Born and surface area solvation (MM-GBSA) energy calculations and molecular dynamic simulations were performed to further characterize the efficacy and stability of the stilbene ligands to the M^pro^ target protein.

## 2. Materials and Methods

### 2.1. Protein Preparation

The crystallographic structure of M^pro^ (PDB Code: 6LU7) was retrieved from the Protein Data Bank and prepared using the protein preparation wizard available in the Maestro v12.3 Schrödinger, LLC, 2020.1 (https://www.schrodinger.com/downloads/releases accessed on 6 June 2022) software package. All missing residues were added, and all ligands, except cofactors and crystallographic water molecules, were removed. H-bonds were adjusted at variable pH, assigned bond order, and the structures protonated according to a pH of 7.0. The protonated structures were minimized using an Optimized Potentials for Liquid Simulations (OPLS) force field.

### 2.2. Ligand Preparation

The structures of 7 stilbene compounds: astringin (1), isorhapontin (2), piceatannol (3), isorhapontigenin (4), resveratrol (5), pinosylvin monomethyl ether (PMME) (6) and pinosylvin (7), and GC376 and N3 as reference compounds, were drawn using Chemdraw (BioDraw ultra, 2014 CambridgeSoft Corporation, accessed on 6th June 2022). All ligands were prepared using the LigPrep module available in Maestro (Schrödinger, LLC, 2020, NY, USA). Energy minimization was carried out using the OPLS-2005 force field.

### 2.3. Molecular Docking Studies

Molecular docking studies were performed using Glide with default parameters, available in Maestro (Glide, Schrödinger, LLC, 2020, NY, USA). First, a binding pocket was located using receptor grid generation, constituting the key residues involved in ligand binding. Molecular docking (XP) calculations were performed using Glide at the binding site of Mpro protein with default parameters. No constraints were applied for all the docking studies. For each compound, multiple poses were attained after the docking process.

### 2.4. Prime Molecular Mechanics-Generalized Born and Surface Area Solvation (MM-GBSA)

For the calculation of binding free energy (ΔG bind) of each ligand docking complex, prime MM-GBSA was applied using the following equation:

ΔGbind = ΔEMM + ΔGsolv + ΔGSA(1)

where ΔEMM is the difference in the minimized energy between the M^pro^-inhibitor complex and the sum of energies of the unliganded M^pro^ and the ligands. ΔGsolv is the difference in MM-GBSA. Solvation energy was applied to analyze the binding free energy decompositions of the M^pro^-ligand complex and the sum of energies for unliganded M^pro^ and the ligand. ΔGSA is the difference in surface area energies for the complex and the sum of the surface area energies for the M^pro^ and ligand when considered individually.

The molecular dynamic simulation was performed based on the receptor-ligand complex obtained from molecular docking. The ligand poses were minimized using the local optimization feature in Prime, OPLS-2005 force field, and a generalized-Born/surface area continuum solvent model was used to calculate the energies of each complex. The ligand strain energy was also considered during the simulation process.

### 2.5. Molecular Dynamics Simulations

The estimation of stability and interaction of the main protease M^pro^ with the most suitable ligands was performed using Maestro-Desmond v12.3 Schrödinger software package (Schrödinger, LLC, 2020, NY, USA) [28]. A molecular dynamic (MD) simulation model was built using a Desmond system builder. Water molecules were added to the system. The protein–ligand complex was kept in an orthorhombic box shape and placed in the center of the box by minimizing the volume in the system builder. The charge of each system was neutralized by addition of Na+ or Cl− ions, and then the system was minimized and pre-equilibrated by using force field Optimized Potentials for Liquid Simulations (OPLS3e), as this produces greater accuracy against performance benchmarks that assess small molecule conformational propensities, solvation, and protein–ligand binding. Each MD simulation was run for a time of 100 ns at a normal pressure and temperature (NPT) ensemble of 300 K temperature and 1.013 bars pressure. The system was set to a relaxed state before simulation by applying the default settings. Protein and ligand structural properties, Root Mean Square Deviation (RMSD) of ligand-protein, and the Root Mean Square Fluctuation (RMSF) for interacting residues with the ligand and the types of interaction and stability of the complex were analyzed.

## 3. Results

### 3.1. Molecular Docking

**Figure 1:**
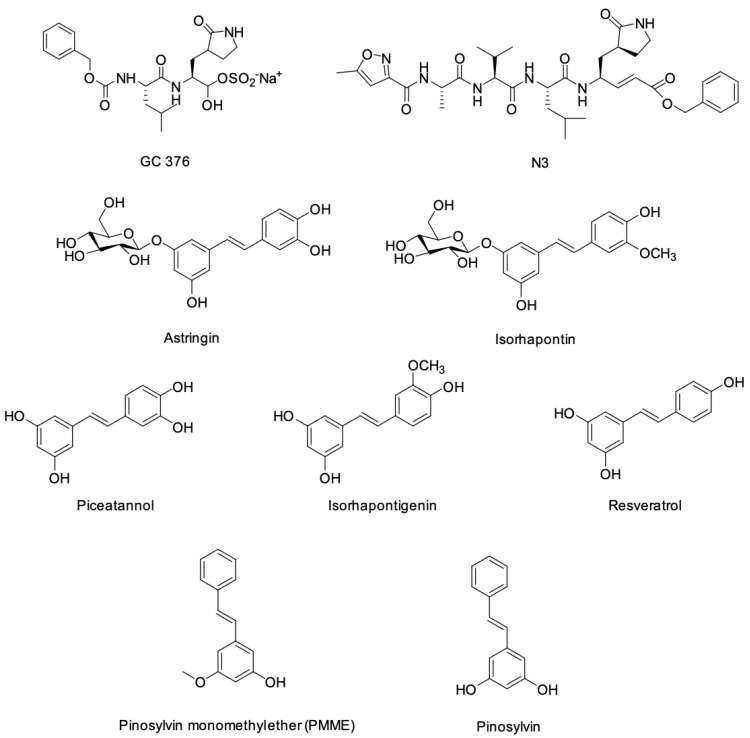
Structures of the reference and stilbene ligands used for the molecular docking studies against Mpro.

The reference ligands and stilbene compounds selected for analysis are detailed in Table 1, and their structures are shown in Figure 1.

**Figure 2:**
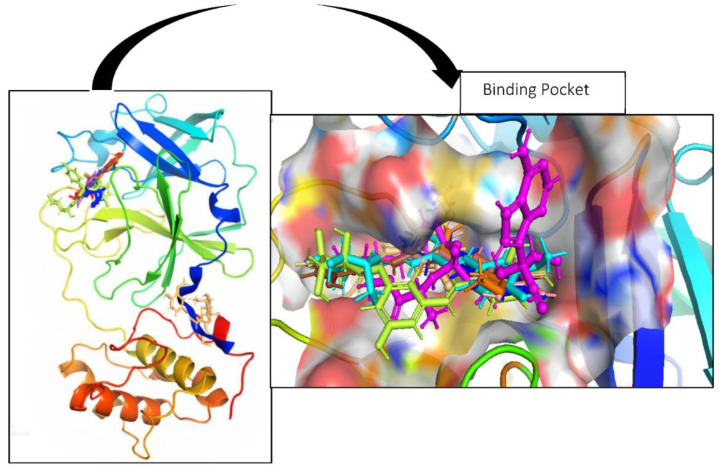
The structure of the Mpro protein (PDB ID: 6LU7) with docked stilbene ligands (compounds 1–7) sharing the same binding pocket.

The stilbene compounds were ranked according to their binding affinities with the SARS-CoV-2 viral enzyme M^pro^ (PDB ID: 6LU7), with GC376 and N3 used as reference ligands. Ligands with a docking score similar to or above that of GC376 and N3 were selected for further molecular docking analysis, as shown in Figure 2.

From within this binding pocket, the binding affinity values for each of the ligands were calculated and ranged from −5.216 (pinosylvin, with the lowest affinity) to −9.319 kcal/mol (astringin, with the highest affinity). These were comparable or superlative to the reference drugs, GC376 and N3, at −6.967 and −6.345 kcal/mol, respectively (refer to Table 2). N3 and GC376 displayed the best minimal glide energy (−0.129 and −0.211 kcal/mol, respectively), a reference score associated with ligand binding free energy, with astringin the next lowest for the ligands. The lipophilic character of all of the stilbenes was relatively comparable and less than GC376 and N3 due to the attachment of a glucoside ring and multiple hydroxyl (OH) groups in the structure (hydrophilic part). However, the glide ligand efficacy, which is a percentage/potency efficiency index (PEI), binding efficiency index (BEI), and surface-binding efficiency index (SEI), of all of the stilbene compounds was higher than that for N3 and GC376, and this can be a useful property to consider for optimization of drug development.

**Figure 3:**
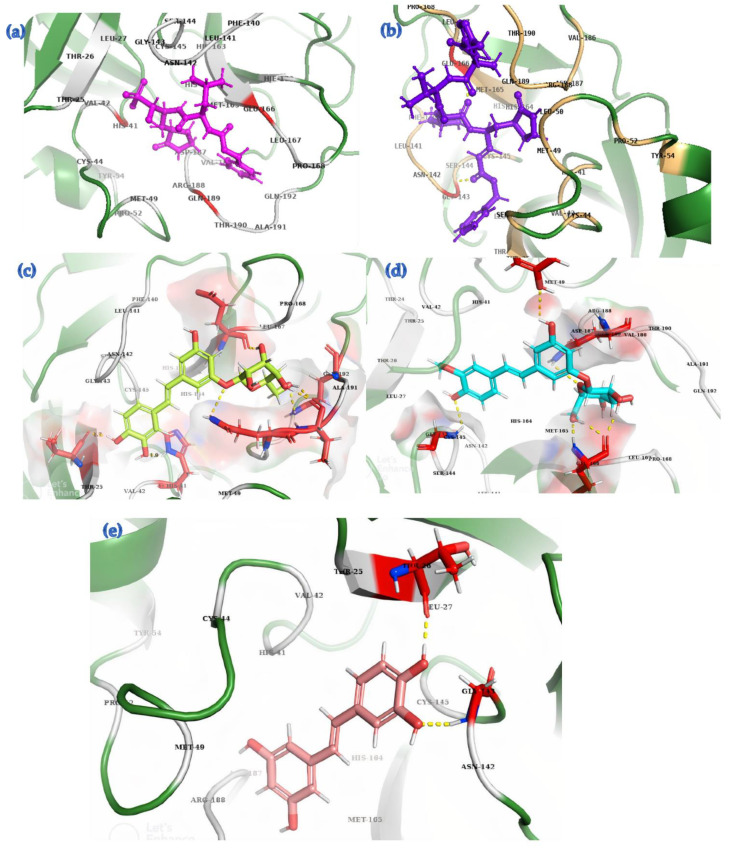

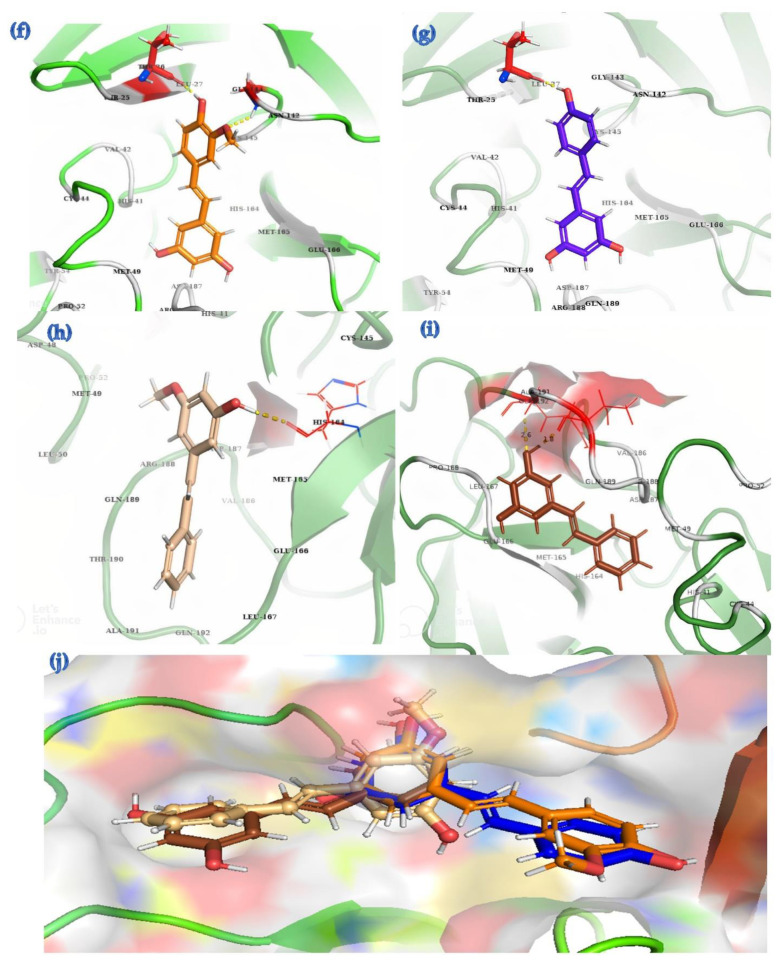
Predicted conformations for ligands within the binding pocket of Mpro. Mpro protein is depicted as a green color string-structure with GC376 (mauve color) (**a**); N3 structure (purple color) (**b**); astringin (Structure 1) (lime green color) (**c**); isorhapontin (Structure 2) (turquoise color) (**d**); piceatannol (Structure 3) (salmon color) (**e**). isorhapontigenin (Structure 4) (orange color) (**f**); resveratrol (Structure 5) (blue color) (**g**); PMME (Structure 6) (wheat color) (**h**); pinosylvin (Structure 7) (Brown color) (**i**); Structures 1–7 within the binding pocket of Mpro (**j**). Red color regions indicate polar residues and grey colors indicate non-polar residues of the Mpro protein.

All seven stilbene ligands and the reference compounds GC376 and N3 have an interaction with His-41, Cys-44, Met-49, His-164, Met-165, Glu-166, Arg-188 and Thr-190, key residues within the active site of M^pro^ as shown in Table 3. All ligands had an interaction with Pro-52 except astringin, all with Cys-145 except pinosylvin, all with Val-186 except N3, all with Gln-189 except isorhapontin, and all with Gln-192 except piceatannol, as detailed in Table 3 and depicted in Figure 3.

**Figure 4:**
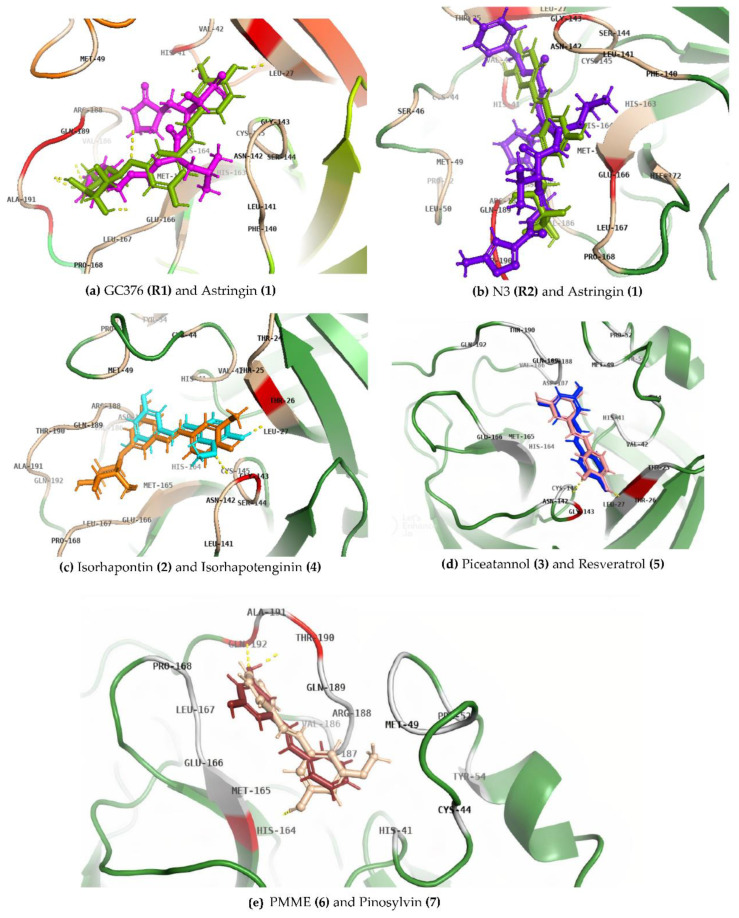
Overlapping docking configurations of ligands bound to M^pro^. GC376 (Reference 1) and Astringin (Structure 1) are shown in (**a**); N3 (Reference 2) and Astringin (Structure 1) in (**b**): Isorhapontin (Structure 2) and Isorhapotenginin (Structure 4) in (**c**); Piceatannol (Structure 3) and Resveratrol (Structure 5) in (**d**); PMME (Structure 6) and Pinosylvin (Structure 7) in (**e**).

Thr-24 of M^pro^ exhibits alkyl (non-polar) interactions only with isorhapontin. Thr-26, Leu-27, Asn-142, and Gly-143 form an interaction with GC376, N3, and all stilbenes except PMME and pinosylvin. Leu-167 and Pro-168 exhibit interactions with GC376, astringin, isorhapontin, PMME, and pinosylvin. His-163 specifically provides a non-polar interaction with GC376, N3, and astringin, as shown in Figure 4.

N3 shows structural stabilization by forming hydrogen bonding with Gly-143 (donor with a C=O) and Glu-166 (acceptor) with the keto group (C=O) of N3 at a distance of 2.0 A and 2.1 A, respectively. GC376 displays hydrogen bonding with His-41, Glu-166, and Gln-189, and it forms a stable complex with M^pro^ due to conventional hydrogen bonds, as detailed in Table 4. Astringin exhibits hydrogen bonding with Gln-189, Gln-192, and Thr-190, and it forms a stable complex with M^pro^ due to conventional hydrogen bonds containing oxygen (-O), hydrogen (-H), and simultaneously as a donor and acceptor with Thr-26 and His-41, respectively. Isorhapontin forms conventional hydrogen bonds with Tyr-54, Gly-143, and Glu-166 at distances of 2.9, 2.7, and 2.0 A, respectively, as detailed in Table 4.

Piceatannol forms conventional H-bonding as well as aromatic H-bonding with Gly-143 and Thr-26 at a distance of 2.5 A and 1.8 A, respectively. Similarly, isorhapontigenin also exhibits conventional and aromatic H-bonding with the same residues at distances of 2.7 A and 1.8 A, respectively. Resveratrol, PMME, and pinosylvin only show conventional H-bonding with Thr-26, His-164, Thr-190, and Gln-192 at distances of 1.8 A, 2.1 A, 1.8 A, and 2.6 A, respectively. The higher binding affinity of astringin than GC376 and N3 is due to a higher degree of hydrogen bonding, as shown in Figure 3. For PMME and pinosylvin, the former ligand has greater binding energy with M^pro^ due to the presence of a methoxy (-OCH3) group as compared to a hydroxyl (OH) group. The binding affinity of piceatannol is higher than resveratrol due to an additional hydroxyl (OH) group in piceatannol, as shown in Figure 4. Isorhapontin has a glucoside side chain in its structure which is the rationale for its higher binding affinity and the stability of the complex, as compared to isorhapontigenin, as shown in Figure 4.

### 3.2. Prime MM–GBSA Simulations

Prime energy calculation analyses were performed to consider the relative binding energies of each ligand to M^pro^. Post-docking energy minimization studies were estimated using Prime molecular mechanics-generalized Born surface area (MM-GBSA). From the results of MM-GBSA studies, a ΔG-binding value was calculated in the range of −111.06 kcal/mol (N3) to −46.95 kcal/mol (resveratrol). These results suggest that GC376, N3, and astringin are relatively highly active against M^pro^, with ΔG-binding values of −77.33 kcal/mol, −111.06 kcal/mol, and −72.78 kcal/mol, respectively, as detailed in Table 5.

### 3.3. MD-Simulations

**Figure 5:**
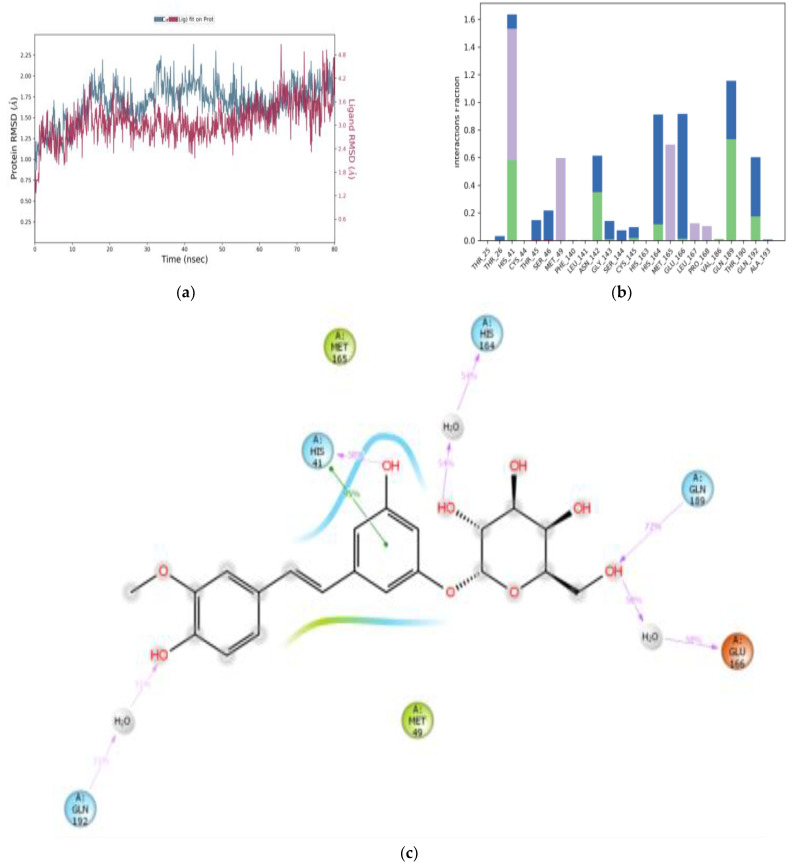
MD simulation studies of astringin (Structure 1) in complex with M^pro^. RMSD plot of protein backbone (Cα) and protein conformational changes during ligand binding (**a**). Interaction fraction plot showing different protein residues that interact with astringin during a 100 ns MD simulation (**b**). Interaction of ligand atoms with the protein residues that occurs for >60% of the simulation time (**c**).

**Figure 6:**
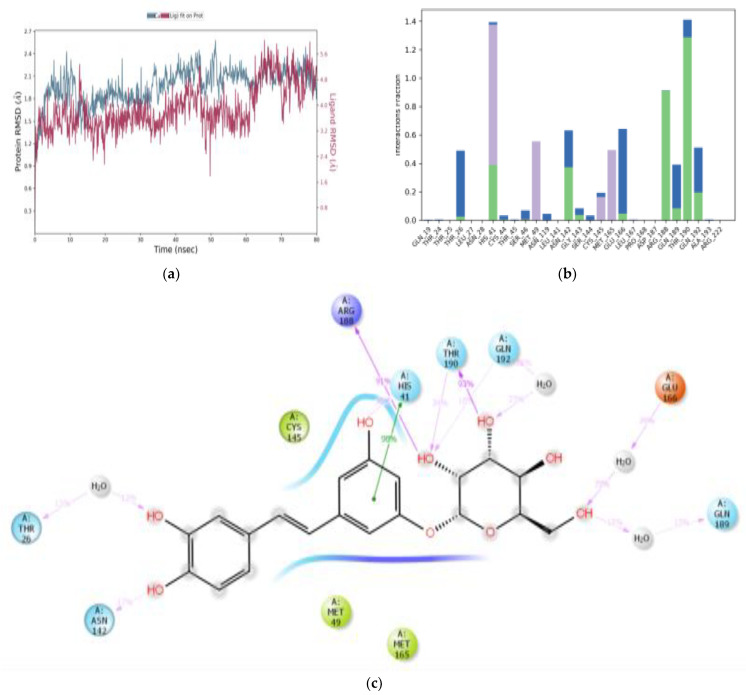
MD simulation studies of isorhapontin (Structure 2) in complex with M^pro^. RMSD plot of protein backbone (Cα) and protein conformational change during ligand binding (**a**). Interaction fraction plot showing different protein residues that interact with isorhapontin during a 100 ns MD simulation (**b**). Interaction of ligand atoms with the protein residues that occurs for >70% of the simulation time (**c**).

**Figure 7:**
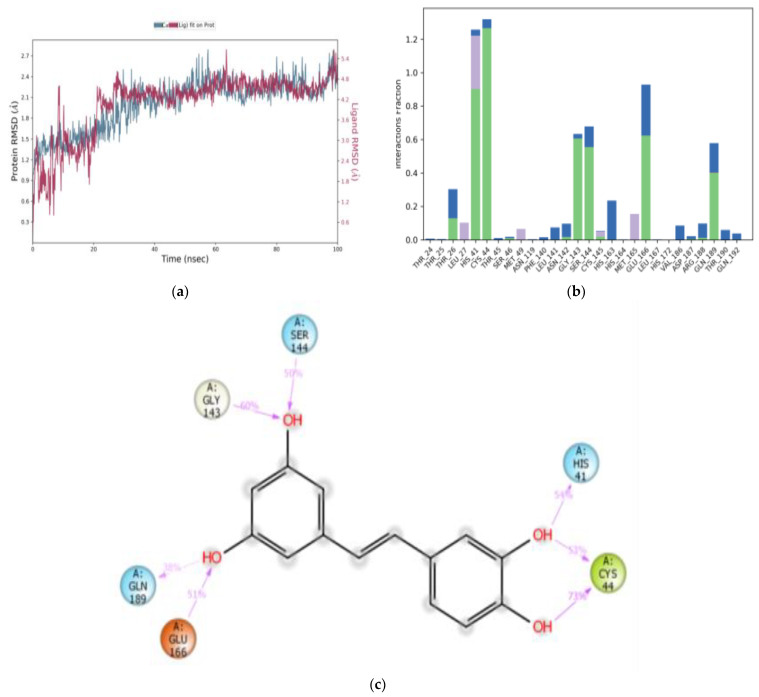
MD simulation studies of piceatannol (Structure 3) in complex with M^pro^. RMSD plot of protein backbone (Cα) and protein conformational change during ligand binding (**a**). Interaction fraction plot showing different protein residues that interact with piceatannol during a 100 ns MD simulation (**b**). Interaction of ligand atoms with the protein residues that occurs for >90% of the simulation time (**c**).

All M^pro^-ligand complexes were subjected to a molecular dynamic (MD) simulation to investigate the dynamic stability of the M^pro^-ligand complexes using the Desmond molecular dynamics system [28,29]. MD simulations were run at NPT for 100 ns, and protein–ligand RMSD plots were generated for each heterodimer binding to M^pro^. The RMSDs and RMSFs of the Cα atoms for each complex were analyzed. The RMSD fingerprints measured the displacement of a selection of astringin, isorhapontin, and piceatannol atoms over this period. Figure 5a, Figure 6a, and Figure 7a show that astringin, isorhapontin, and piceatannol, respectively, all formed stable complexes with the protein during the simulation time. The highest fluctuations were observed in a few regions, ranging from 5–10 ns, 30–40 ns, and 50–60 ns for astringin, as shown in Figure 5a, 30 to 50 ns for isorhapontin, as shown in Figure 6a, and with slight fluctuation observed with piceatannol, ranging from 3–8 ns and 25–30 ns of the simulation period, as shown in Figure 7a.

The interaction of the protein with astringin was also recorded as interaction fraction plots for the heterodimer binding to the protein during the simulation. With regards to the stability of the complex of M^pro^ with astringin, His-41 forms 100% stable hydrophobic interactions and hydrogen bonds and water bridges throughout the simulation time. Met-49 and Met-165 are involved in hydrophobic interactions for 65% of the simulation time. Similarly, Asn-142, Gln-189, Glu-166, and Gln-192 form hydrogen bonds and water bridges for 80% of the simulation time (Figure 5b). Thr-45, Ser-46, Gly-143, Ser-144, Cys-145, Leu-167, and Pro-168 are important for stabilizing the ligand-protein complex, as shown in Figure 5c, according to stability studies of >60% of the simulation time. Similarly, for isorhapontin, Asn-142, Gln-192, and Glu-166 form hydrogen bonds and water bridges for 65% of the simulation time. Thr-26, Cys-44, Ser-46, Asn-119, Gly-143, Ser-144, and Cys-145 are important for stabilizing the ligand-protein complex, as shown in Figure 6b. For the assessment of interacting residues, isorhapontin forms 95% stable hydrophobic interaction and 58% stable hydrogen bonds with His-41, 72%, 58%, and 54% stable hydrogen bonding with Gln-189, Glu-166, and His-164, respectively, as shown in Figure 6c. The interaction of piceatannol with M^pro^ was also recorded as interaction fraction plots for the heterodimer binding to the protein during the simulation. Cys-44 forms hydrogen bonds (as acceptor) with the ligand almost 100% of the time, and His-41 also forms 100% hydrophobic interactions, hydrogen bonds, and water bridges for almost 80% of the simulation time. Similarly, Cys-44, Gly-143, Ser-144, Glu-166, and Gln-189 make simultaneous donor-acceptor hydrogen bonding and water bridges for 80% of the simulation time. Leu-27, Met-49, Leu-141, Asn-142, Cys-145, His-163, Met-165, Arg-188, Thr-190, and Gln-192 are other amino acids important for stabilizing the piceatannol-protein complex as shown in Figure 7b. For the assessment of interacting residues, Cys-44 and Gly-143 form 73% and 60% stable hydrogen bonds, respectively, as compared to His-41, Glu-166, Gln-189, and Ser-144 with 50% of the simulation time, as shown in Figure 7c. According to the MD-simulation studies, all the ligands are stable in the binding pocket of M^pro^.

**Figure 8:**
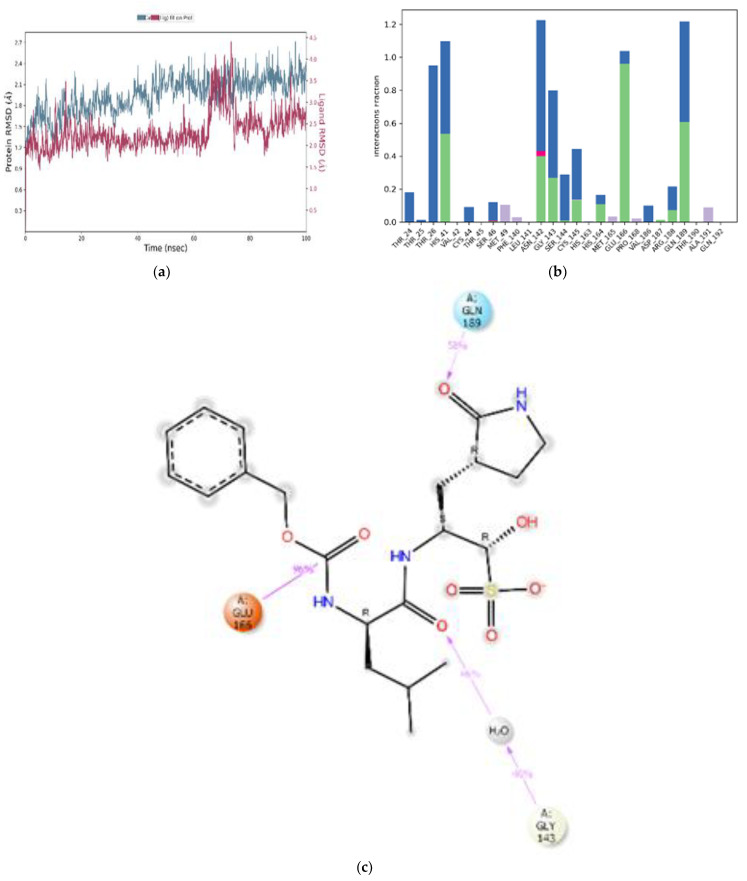
MD simulation studies of GC376 (Reference 1) in complex with M^pro^. RMSD plot of protein backbone (Cα) and protein conformational change during ligand binding (**a**). Interaction fraction plot showing different protein residues that interact with GC376 during a 100 ns MD simulation (**b**). Interaction of ligand atoms with the protein residues that occurs for >70% of the simulation time (**c**).

**Figure 9:**
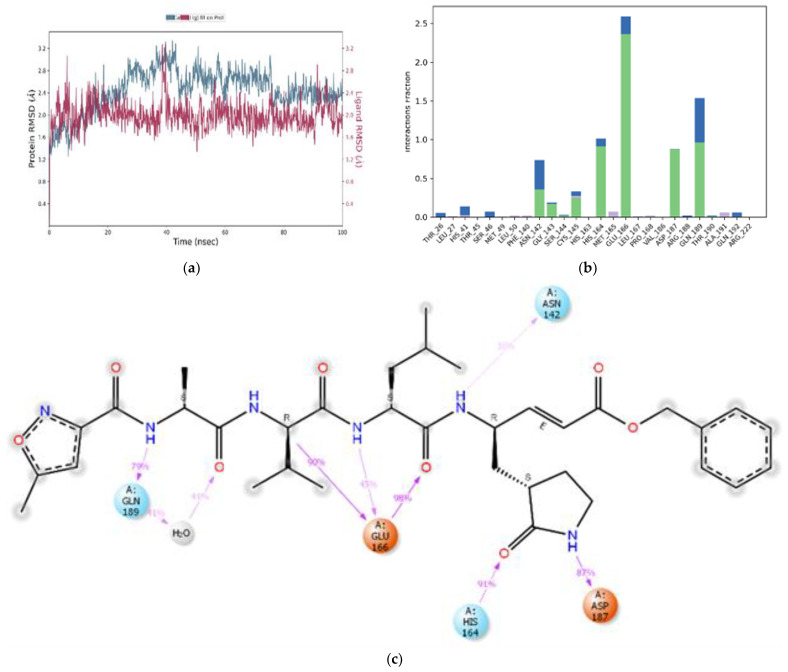
MD simulation studies of N3 (Reference 2) in complex with Mpro. RMSD plot of protein backbone (Cα) and protein conformational change during ligand binding (**a**). Interaction fraction plot showing different protein residues that interact with N3 during a 100 ns MD simulation (**b**). Interaction of ligand atoms with the protein residues that occurs for >70% of the simulation time (**c**).

The dynamic stability of the reference ligands GC376 and N3 was also ascertained using MD simulation studies. Here, the RMSD plot for GC376 showed fluctuations between 5–10 ns and 18–68 ns. The complex then showed configurational complementarity between 69–86 ns and eventually, the compound drifted from the M^pro^ binding pocket at 87–100 ns, as shown in Figure 8a. By contrast, N3 showed fluctuations between 20–38 ns and 40–92 ns of the simulation time, as depicted in Figure 9a. Furthermore, the interaction fraction plot of GC376 with target protein M^pro^ indicates that the compound forms water bridges, hydrogen, and ionic bonds with Asn-142 for 100% of the simulation time. His-41 forms hydrogen bonds and water bridges for 100% of the simulation time and Glu-166 forms stable hydrogen bonds for 96% of the simulation time and Gln-189 for 58%, as shown in Figure 8b. Additionally, Gly-143 forms hydrogen bonds and water bridges with GC376 for 46% of the simulation time (Figure 8b). Thr-26, Cys-145, Ser-144, Thr-24 and Arg-188 are some of the other contributing amino acids that form a stable complex with GC376 (Figure 8c). From the interaction fraction plot of N3, Glu-166 was found to form hydrogen bonds and water bridges for 100% of the simulation period, whereas Gln-189 formed hydrogen bonds for 79% and water bridges for 41% of the simulation time. His-164 formed hydrogen bonds for 91% of simulation time and Asp-187 for 87% of simulation time as shown in Figure 9b. Asn-142 of M^pro^ forms hydrogen bonds for 30% of the simulation time, and Cys-145, Gly-143 and His-41 are some of the other amino acid residues contributing to the N3-M^pro^ binding (Figure 9c).

## 4. Discussion

In this study, an in silico strategy was employed to consider the molecular docking, prime MM-GBSA, and MD simulations for several stilbene ligands binding to the SARS-CoV-2 protein, M^pro^. After molecular docking studies, deduction of the most favorably docked conformations of the studied ligands was undertaken and revealed the involvement of a number of essential residues that provided polar and non-polar binding interactions to support ligand binding within the M^pro^ binding pocket, especially His-41, Cys-44, Met-49, His-164, Met-165, Glu-166, Val-188, and Thr-190. These residues provide binding and stabilization to the stilbene ligands via hydrogen bonding, hydrophobic interactions, and π-π interactions. MM-GBSA calculations provided further insight into the free energy associated with the binding of the stilbene ligands to M^pro^, and the ΔG value for astringin, isorhapontin, and piceatannol were similar or superlative to the reference ligands, GC376 and N3, and with improved binding performance than the other stilbene ligands isorhapontigenin, resveratrol, PMME, and pinosylvin. Furthermore, the stilbene ligands capable of relatively strong binding to M^pro^ generated affinity values similar to those calculated for drugs specifically optimized for binding to M^pro^ using computer-aided drug design [19]. To further scrutinize ligand binding, MD simulations provided an insight into the dynamic behavior of the ligand-protein complex, and a series of snapshots of astringin, isorhapontin, and piceatannol binding to M^pro^ confirmed the stability of ligand binding and provided a depiction of the protein residues that contribute to this binding.

Collectively, stilbenes represent a chemically diverse group with biological activities in addition to their inherent antioxidant capabilities [22,25,26]. The stilbene skeleton may provide a useful skeleton for the design of natural inhibitors of drugs of M^pro^ [27], and this was evaluated in this study for a number of stilbene compounds that can be extracted directly from natural sources [22,23,24].

Natural stilbenes confer plant disease resistance via their antimicrobial activities [30], but their toxicity to humans has been less well-established. Natural plant inhibitors may provide therapeutic agents with perceived or actual lower toxicity than commercial synthetic drugs [31,32]. Furthermore, for nations with a developing healthcare system, plant-based treatments may be more accessible than commercial drugs.

The reference ligands GC376 and N3 exhibit binding and inhibitory activity against M^pro^ and can suppress viral replication [17,18,33,34]; hence, ligands with similar or even more potent affinities for M^pro^ than these drugs may also prove useful as M^pro^ inhibitors. However, this study only represents a first step in the identification of natural inhibitors of M^pro^, and further in vitro toxicity and efficacy testing of these selected stilbene compounds for their enzymatic inhibition of M^pro^ is now required. Most notably, the most promising candidate structures (astringin, isorhapontin, and piceatannol) will need to be assessed before their potential utilization as a treatment option in vivo to counter replication of the SARS-CoV-2 virus. However, since these stilbene ligands currently exhibit relatively strong binding affinities to M^pro^, there is also scope for manipulation of their chemical backbones to further optimize target binding and potential improvement of drug efficacy. The additional benefit of utilizing these natural stilbenes is that some display known anti-inflammatory activities [25,35,36,37], and this may be useful to counter the potentially life-threatening cytokine storm that can be induced after infection with SARS-CoV-2 virus [38,39]. Lastly, the dietary availability of certain stilbenes, including piceatannol and resveratrol that are present in fruits and berries such as grapes and blueberries [40,41], infers that dietary choices that include these natural functional foods could provide protection against or at least mitigate the effects of the SARS-CoV-2 virus.

## Figures and Tables

**Table 1:** Reference and stilbene compounds used for the molecular docking studies against M^pro^.

Ligand	Name	Molecular Formula	Molecular Weight
Reference 1	GC376	C_21_H_30_N_3_NaO_8_S	507.50 g/mol
Reference 2	N3	C_35_H_48_N_6_O	680.80 g/mol
1	Astringin	C_20_H_22_O_9_	406.40 g/mol
2	Isorhapontin	C_21_H_24_O_9_	420.40 g/mol
3	Piceatannol	C_14_H_12_O_4_	244.24 g/mol
4	Isorhapontigenin	C_15_H_14_O_4_	258.27 g/mol
5	Resveratrol	C_14_H_12_O_3_	228.25 g/mol
6	Pinosylvin monomethyl ether (PMME)	C_15_H_14_O_2_	226.27 g/mol
7	Pinosylvin	C_14_H_12_O_2_	212.24 g/mol

**Table 2:** Molecular docking results and interaction details for the reference and stilbene compounds.

Ligand	Name	Binding Affinity (XP-Score)	Glide Score	Glide Energy	Lipophilic Score	XP-Hbond	Glide-Ligand Efficacy
Reference 1	GC376	−6.976	−6.976	−56.004	−8.946	−1.353	−0.211
Reference 2	N3	−6.345	−6.345	−71.968	−19.024	−1.300	−0.129
1	Astringin	−9.319	−9.319	−48.561	−4.030	−1.440	−0.321
2	Isorhapontin	−8.166	−8.166	−45.315	−5.525	−3.501	−0.272
3	Piceatannol	−6.291	−6.291	−35.995	−1.804	−2.124	−0.350
4	Isorhapontigenin	−5.877	−5.877	−35.997	−2.729	−0.960	−0.309
5	Resveratrol	−5.753	−5.753	−33.736	−1.805	−0.587	−0.338
6	PMME	−5.460	−5.460	−31.291	−2.535	−0.303	−0.321
7	Pinosylvin	−5.216	−5.216	−31.202	−1.804	−0.597	−0.326

**Table 3:** Polar and non-polar interactions of the reference and stilbene compounds with amino acids within the M^pro^ binding pocket.

	Name	Interaction Type	Residues
Reference 1	GC376	Polar	His-41, Gln-89, Glu-166.
		Non-polar	Thr-25, Thr-26, Leu-27, Val-42, Cys-44, Met-49, Pro-52, Tyr-54, Phe-140, Leu-141, Asn-142, Gly-143, Ser-144, Cys-145, His-163, His-164, Met-165, Phe-181, Val-186, Asp-187, Arg-188, Gln-189, Thr-190, Gln-192
Reference 2	N3	Polar	Gly-143, Glu-166.
		Non-polar	Thr-25, Thr-26, Leu-27, His-41, Val-42, Cys-44, Ser-46, Met-49, Leu-50, Pro-52, Phe-140, Leu-141, Asn-142, Ser-144, Cys-145, His-163, His-164, Met- 165, Leu-167, Asp-187, Arg-188, Gln-189, Thr-190, Ala-191, Gln-192.
1	Astringin	Polar	Thr-26, His-41, Gln-189, Thr-190, Gln-192, Glu-166.
		Non-polar	Thr-25, Leu-27, Val-42, Cys-44, Met-49, Phe-140, Leu-141, Asn-142, Gly-143, Ser-144, Cys-145, His-163 His-164, Met-165, Leu-167, Pro-168, Val-186, Arg-188, Ala-191.
2	Isorhapontin	Polar	Tyr-54, Gly-143, Glu-166.
		Non-polar	Thr-24, Thr-25, Thr-26, Leu-27, His-41, Val-42, Cys-44, Met-49, Pro-52, Leu-141, Asn-142, Ser-144, Cys-145, His-164, Met-165, Leu-167, Pro-168, Val-186, Asp-187, Arg-188, Thr-190, Ala-191, Gln-192.
3	Piceatannol	Polar	Thr-26, Gly-143.
		Non-polar	Thr-25, Leu-27, His-41, Val-42, Cys-44, Met-49, Pro-52, Tyr-54, Asn-142, Ser-144, Cys-145, His-164, Met-165, Glu-166, Val-186, Asp-187, Arg-188, Gln-189, Thr-190.
4	Isorhapontigenin	Polar	Thr-26, Gly-143.
		Non-polar	Thr-25, Leu-27, His-41, Val-42, Cys-44, Met-49, Pro-52, Tyr-54, Asn-142, Ser-144, Cys-145, His-164, Met-165, Glu-166, Val-186, Asp-187, Arg-188, Gln-189, Thr-189, Gln-192.
5	Resveratrol	Polar	Thr-26.
		Non-polar	Thr-25, Leu-27, His-41, Val-42, Cys-44, Met-49, Pro-52, Asn-142, Gly-143, Cys-145, His-164, Met-165, Glu-166, Val-186, Asp-187, Arg-188, Gln-189, Thr-190, Gln-192.
6	PMME	Polar	His-164.
		Non-polar	His-41, Cys-44, Asp-48, Met-49, Leu-50, Pro-52, Tyr-54, Cys-145, Met-165, Glu-166, Leu-167, Pro-168, Val-186, Asp-187, Arg-188, Gln-189, Thr-190, Ala-191, Gln-192.
7	Pinosylvin	Polar	Thr-190, Gln-192.
		Non-polar	His-41, Cys-44, Met-49, Pro-52, Tyr-54, His-164, Met-165, Glu-166, Leu-167, Pro-168, Val-186, Asp-187, Arg-188, Gln-189, Ala-191.

**Table 4:** Distance parameters of hydrogen bonds derived from molecular docking of reference and stilbene ligands with M^pro^.

Ligand Structure	Name	Hydrogen Bonding			
		Bonding Type	Protein	Ligand Element	Distance (A)
			Interacting Amino Acids	Interacting Atom or Ring	
Reference 1	GC376	Conventional H-bond	His-41	O	2.1
			Glu-166	O	1.9
			Gln-189	O	2.9
Reference 2	N3	Conventional H-bond	Gly-143	O	2.0
			Glu-166	O	2.1
1	Astringin	Conventional H-bond	Thr-26	OH−	1.7
			His-41	O−	1.9
			Gln-189	O−	2.8
			Gln-192	O−	2.5
			Thr-190	OH−	1.9
				OH−	2.0
2	Isorhapontin	Conventional H-Bond	Tyr-54	OH−	2.9
			Gly-143	O	2.7
			Glu-166	O	2.1
				OH−	2.0
				OH−	2.0
3	Piceatannol	Conventional H-Bond	Gly-143	O	2.5
		Aromatic H-bond	Thr-26	H−	1.8
4	Isorhapontigenin	Conventional H-Bond	Gly-143	O	2.7
		Aromatic H-bond	Thr-26	OH−	1.8
5	Resveratrol	Conventional H-Bond	Thr-26	OH−	1.8
6	PMME	Conventional H-bond	His-164	OH−	2.1
7	Pinosylvin	Conventional H-bonding	Thr-190	OH−	1.8
			Gln-192	O	2.6

**Table 5:** Prime MM-GBSA results for reference and stilbene compounds binding to M^pro^.

Ligand	Name	Prime Energy	Ligand Efficiency	Ligand Efficiency ln	ΔG Bind	ΔG Bind Coulomb	ΔG Bind Solv.GB	ΔG Bind (NS)	ΔG Bind (NS) Coulomb	ΔG Bind (NS) Solv. GB
Reference 1	GC376	−13112.50	−2.343	−17.199	−77.33	−5.26	23.29	−88.03	−7.73	24.93
Reference 2	N3	−13184.14	−2.267	−22.703	−111.06	−15.77	28.43	−128.12	−21.06	30.29
1	Astringin	−13013.2	−2.510	−16.665	−72.78	−32.12	23.10	−76.13	−31.33	22.71
2	Isorhapontin	−12998.9	−1.881	−12.821	−56.43	−7.86	28.21	−71.60	−21.77	32.63
3	Piceatannol	−13026.6	−2.782	−12.872	−50.08	−15.51	20.12	−52.60	−14.61	19.75
4	Isorhapontigenin	−13016.9	−2.641	−12.722	−50.18	−14.78	19.22	−53.93	−14.11	19.06
5	Resveratrol	−13017.0	−2.762	−12.249	−46.95	−10.10	16.37	−49.09	−9.53	16.41
6	PMME	−13013.8	−3.315	−14.700	−56.35	−7.11	15.17	−61.41	−5.78	14.61
7	Pinosylvin	−13007.9	−2.984	−12.654	−47.74	−7.29	14.44	−52.62	−6.62	14.16

Calculations of binding-free energies (kcal/mol) were undertaken in which MM-GBSA ΔG Bind = Complex − Receptor − Ligand and MM-GBSA ΔG Bind (NS) = Complex − Receptor (from optimized complex) − Ligand (from optimized complex) = MM-GBSA ΔG Bind − Receptor Strain − Ligand Strain. NS, no strain, the binding energy without considering for the receptor and ligand conformational changes needed for the formation of the complex.

## Data Availability

Not applicable.
